# Outcomes of a Modified Envelope Flap Technique With Connective Tissue Graft and Enamel Matrix Derivative for Multiple Gingival Recessions: A Case Report

**DOI:** 10.7759/cureus.76990

**Published:** 2025-01-06

**Authors:** Salvatore L La Terra, Domenico Travaglini, Gianluigi Caccianiga, Faisal Alzahrani, Faris M. Alabeedi

**Affiliations:** 1 Faculty of Natural Health Science, Regenerative Cellular Therapy, Selinus University, London, GBR; 2 Periodontology, Oral Surgery, Private Dental Clinic, Rome, ITA; 3 Oral Surgery, Private Dental Clinic, Modena, ITA; 4 Translational Medicine, University of Ferrara, Ferrara, ITA; 5 Oral Surgery, Ulster University College of Medicine and Dentistry, Birmingham, GBR; 6 Oral Surgery, Ministry of Defense, Royal Armed Forces Medical Services, Riyadh, SAU; 7 Faculty of Dentistry, Maxillofacial Surgery and Diagnostic Science, Najran University, Najran, SAU

**Keywords:** connective tissue graft, ctg, emd, enamel matrix derivative, gingival recession, keratinized tissue width, mucogingival surgery, multiple recessions, periodontal plastic surgery, root coverage

## Abstract

Gingival recession is a prevalent condition with significant aesthetic and functional consequences. This case report is about a 36-year-old female who presented with multiple Miller's Class I gingival recession defects affecting teeth from 22 to 25. A modified envelope flap technique was utilized, incorporating connective tissue graft (CTG) harvested from the palate and supplemented with enamel matrix derivatives (EMD). The treatment resulted in significant root coverage in all treated sites. The patient reported high satisfaction with the aesthetic and functional improvements. This case report demonstrates the successful application of a combined approach utilizing a modified envelope flap, CTG, and EMD for the treatment of multiple gingival recessions. The findings suggest the potential of this technique for achieving predictable and favorable outcomes in similar cases.

## Introduction

Gingival recession is a common periodontal condition characterized by the apical displacement of the gingival margin relative to the cement-enamel junction (CEJ) [1-3]. It may affect a single tooth, a group of teeth, or the entire dentition [4], with buccal surfaces being more commonly and severely affected, particularly as age advances [5,6]. Normally, the gingival margin follows a scalloped contour, positioned 1-2 mm coronally to the CEJ [7]. However, apical migration of the gingival margin can lead to esthetic concerns, increased susceptibility to root caries, dentin hypersensitivity (DH), cervical root abrasions, erosions, and plaque retention [8-10]. This can significantly impact oral health, aesthetics, and overall quality of life [11].

Treatment options for gingival recession primarily involve various surgical techniques aimed at root coverage. These procedures focus on the buccal surface with minimal or no interproximal attachment loss [12] and aim to restore esthetics [13], reduce hypersensitivity [14], and increase gingival width and thickness (GT) [15]. Considering these, the American Academy of Periodontology (AAP) replaced the term ‘mucogingival surgery’ with ‘periodontal plastic surgery’ [3]. Furthermore, Cairo et al. introduced the Root Coverage Esthetic Score, comprising five variables (gingival margin level, marginal contour, soft tissue texture, mucogingival junction position, and gingival color) to reliably assess esthetic outcomes following periodontal plastic surgery [16,17].

Despite advancements, variability in root coverage outcomes remains evident, and achieving 100% coverage continues to be challenging [1,14,18,19]. Additionally, the influence of specific periodontal clinical parameters on root coverage outcomes is not fully understood. Studies have identified factors such as tooth rotation, tooth extrusion (with or without occlusal abrasion), and reduced papilla height as limiting factors for root coverage [12,20].

This case report presents clinical and aesthetic outcomes of a modified surgical technique with connective tissue graft (CTG) and enamel matrix derivative (EMD) for multiple gingival recessions in the maxillary arch. It also highlights the treatment planning and surgical approach, providing valuable insights into managing multiple gingival recession defects and demonstrating the effectiveness of a combined surgical technique.

## Case presentation

Patient information

A 36-year-old female patient came for a consultation with a chief complaint of an unaesthetic appearance when smiling. She sought treatment for multiple gingival recession defects. The patient was a non-smoker in good general health, with no contraindications for dental treatment. Her dental history included previous restorative treatments performed by another practitioner, but she had not received any periodontal therapy in the six months before the presentation. No relevant psychosocial or family history was reported that might have influenced her oral health or treatment outcomes.

Clinical findings

Periodontal Evaluation

**Figure 1:**
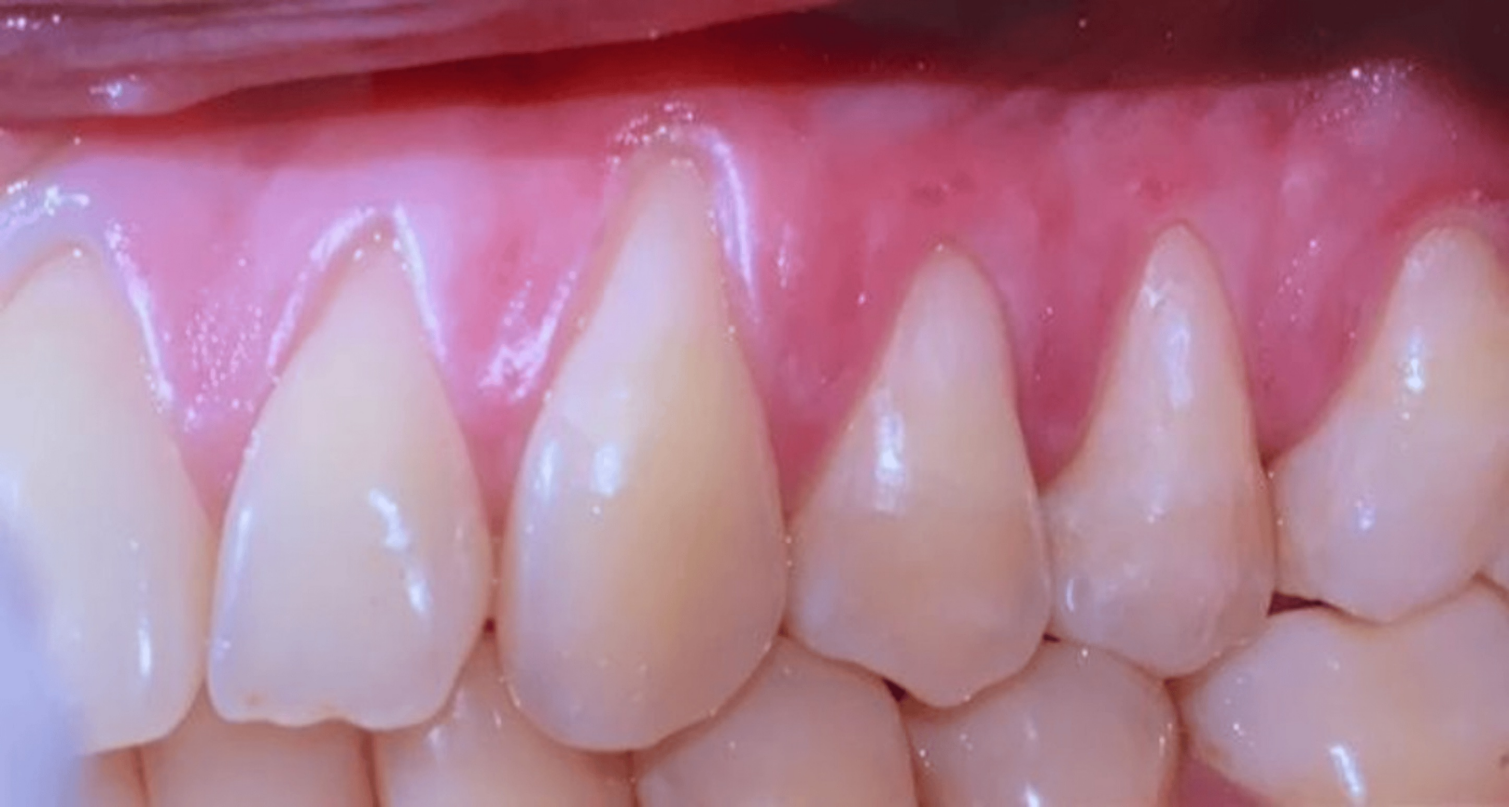
Baseline clinical situation. Preoperative view: maxillary left quadrant, buccal view of the multiple recession defects extending from element 22 to 25.

The patient demonstrated good oral hygiene (OH) with a full-mouth plaque score (FMPS) of 22% and bleeding on probing (BoP) below 10%. A thorough periodontal evaluation revealed the presence of multiple Miller's Class I gingival recession defects affecting teeth 22-25. The baseline clinical measurements for teeth 22-25 revealed a uniform probing depth (PD) of 1 mm across all teeth, with gingival recession ranging from 1.5 to 3.5 mm. Clinical attachment level (CAL) ranged from 2.5 to 4.5 mm. Both plaque index (PI) and BoP were 0% for all teeth. Gingival thickness (GT) varied from 0.8 to 1.4 mm, and keratinized tissue width (KTW) ranged from 1.5 to 2.5 mm. There were no clinical or radiological signs of periodontitis, nor did the patient report any parafunctional habits. Figure 1 illustrates the clinical situation at baseline.

Diagnostic Assessment

A comprehensive oral and periodontal examination was conducted to evaluate gingival health, bone levels, and potential underlying pathology. The patient exhibited positive tactile and air-blast sensitivity, with dentin hypersensitivity and esthetics evaluated using the visual analog scale (VAS) analysis [21]. Based on the clinical examinations, the patient was diagnosed with multiple Miller's Class I gingival recession defects affecting teeth 22-25. From the data available, the recessions were attributed to traumatic toothbrushing, particularly due to the thin biotype of the gingiva in the affected areas. The prognosis for this case was considered to be good based on factors such as the patient's age and OH practices, the severity of the gingival recession, the absence of underlying systemic diseases, and patient compliance with treatment.

Therapeutic intervention

The decision-making process was based on a careful anatomic analysis of the recession defects in order to allow a higher probability of complete root coverage. The approach for predicting the extent of root coverage involved calculating the ideal height of the interdental papilla, which served as a guide for treatment planning [12,20,22].

The treatment plan consisted of initial therapy, followed by periodontal plastic surgery employing coronally advanced flap (CAF) for multiple gingival defects (from 22 to 25) plus CTG and EMD (Straumann® Emdogain®). In this respect, palatal area evaluation at the donor site was performed; the overall procedure was explained to the patient, and informed consent was obtained.

Initial therapy

The patient received basic periodontal treatment with scaling and root planing and instructions for a proper, non-traumatizing tooth brushing technique (roll method) with a soft toothbrush to ensure maintenance before, during, and after therapy. After two weeks, the patient was recalled to assess OH and gingival status.

Surgical procedures and post-operative care

*Surgical Approach and Techniqu*e

A modified design of the envelope type of CAF without any vertical releasing incisions, proposed by Zucchelli et al. [23], for treating multiple recession defects [21], was adopted in this case in conjunction with CTG and EMD. This technique allows simultaneous treatment of multiple adjacent recessions, thus reducing the number of surgeries and overall morbidity.

Local Anesthesia and Surgical Incisions 

**Figure 2:**
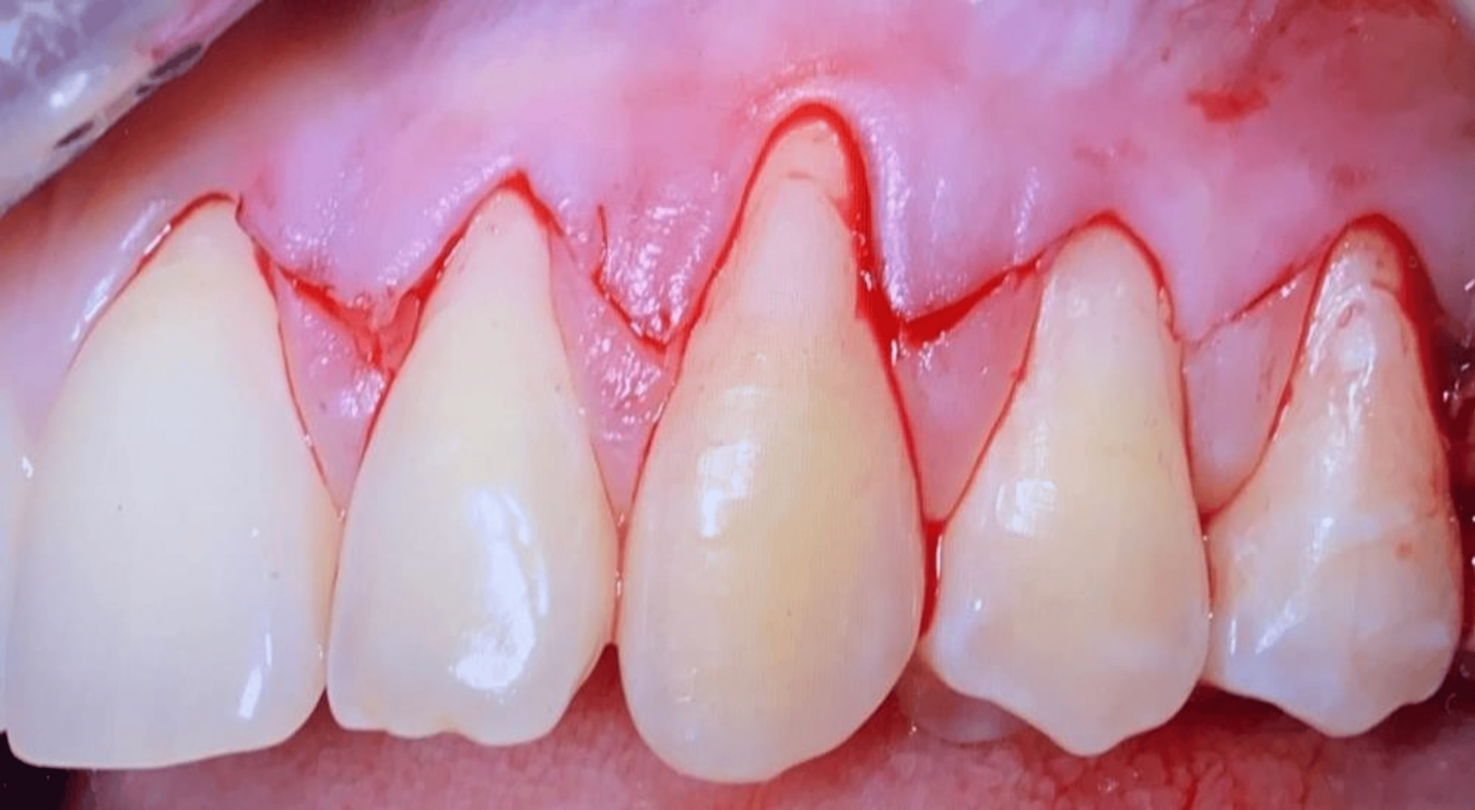
Surgical incision design.

After local anesthesia (articaine hydrochloride with 1:200,000 adrenaline) was administered, surgical incisions were made in the 22-25 region using a No. 15c blade. The flap horizontal incision consisted of oblique submarginal incisions in interdental areas and continued with the intrasulcular incision at the recession defects. The interdental submarginal incisions and the intrasulcular incisions at the mesial/distal margins of the recession defects formed the surgical papillae of the envelope flap. The oblique incisions were made on the proximal sides of the tooth, at a distance equal to the recession depth plus 1/2 mm from the anatomic papilla tip. Figure 2 illustrates the surgical incision design. The envelope flap was raised using a split-full-split approach in the coronal-apical direction.

Flap Elevation and Mobilization

**Figure 3:**
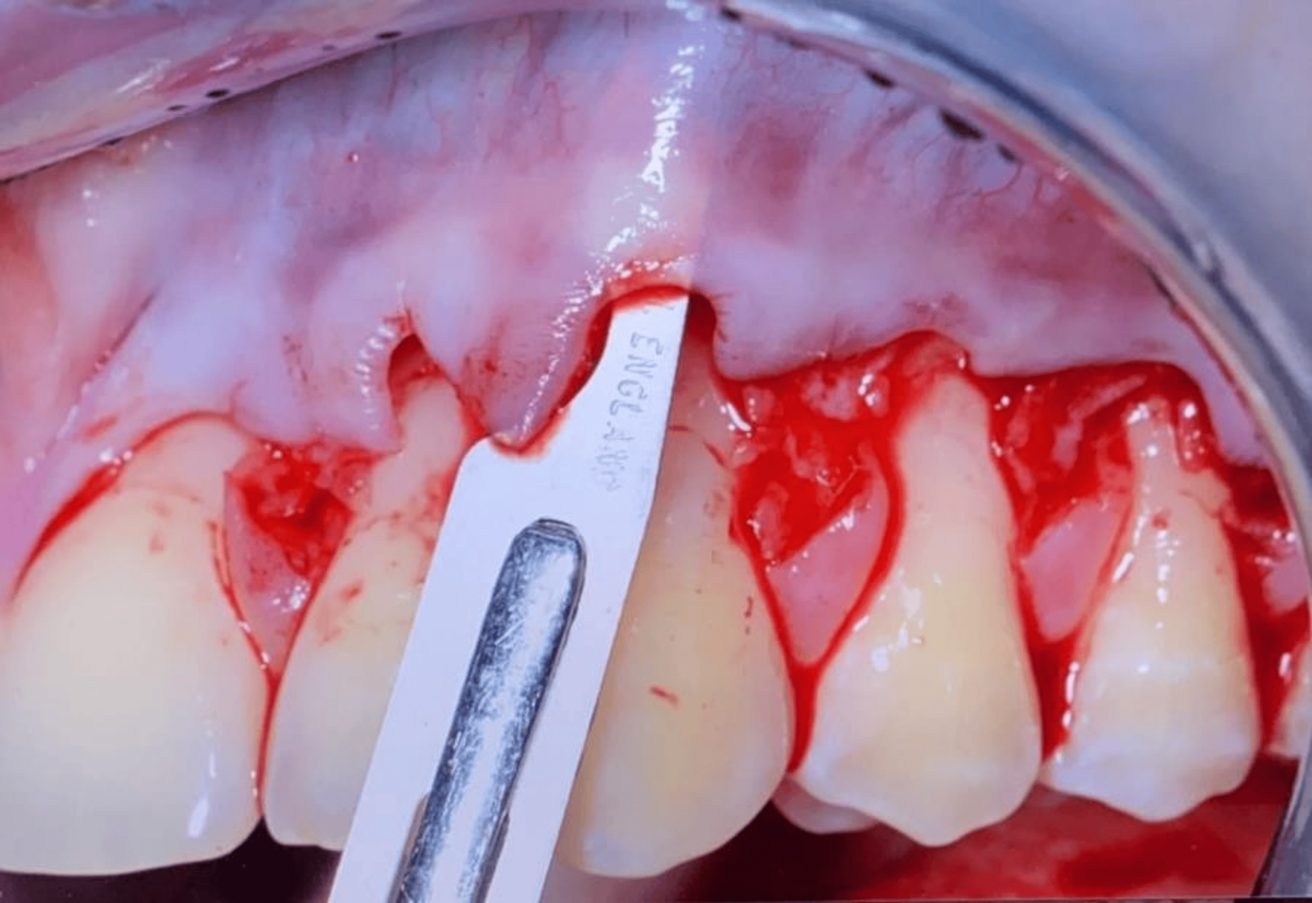
Flap mobilization and coronal advancement technique.

Sharp dissection was employed to mobilize the flap within the vestibular lining mucosa. The surgical papillae were elevated with a split-thickness approach, maintaining blade orientation parallel to the teeth. This split elevation terminated at the level of an imaginary line connecting the probable sulcular areas of the two adjacent recessions. Gingival tissue apical to the root exposure was raised to full thickness to provide more thickness to the portion of the flap critical for root coverage. This full-thickness elevation was performed with a small periosteal elevator inserted into the sulcus, and it terminated once 3-4 mm of bone was denuded apical to the bone dehiscence. To release residual muscle tension, flap dissection was extended apically to the mucogingival junction. A split-thickness approach was utilized, enabling coronal flap advancement. Throughout the procedure, the surgical blade was kept parallel to the flap and pulled the lip for identifying muscular insertion, as shown in Figure 3. The thin-lying mucosa allowed for controlling blade movement.

De-epithelialization of Papilla and Flap Advancement

**Figure 4:**
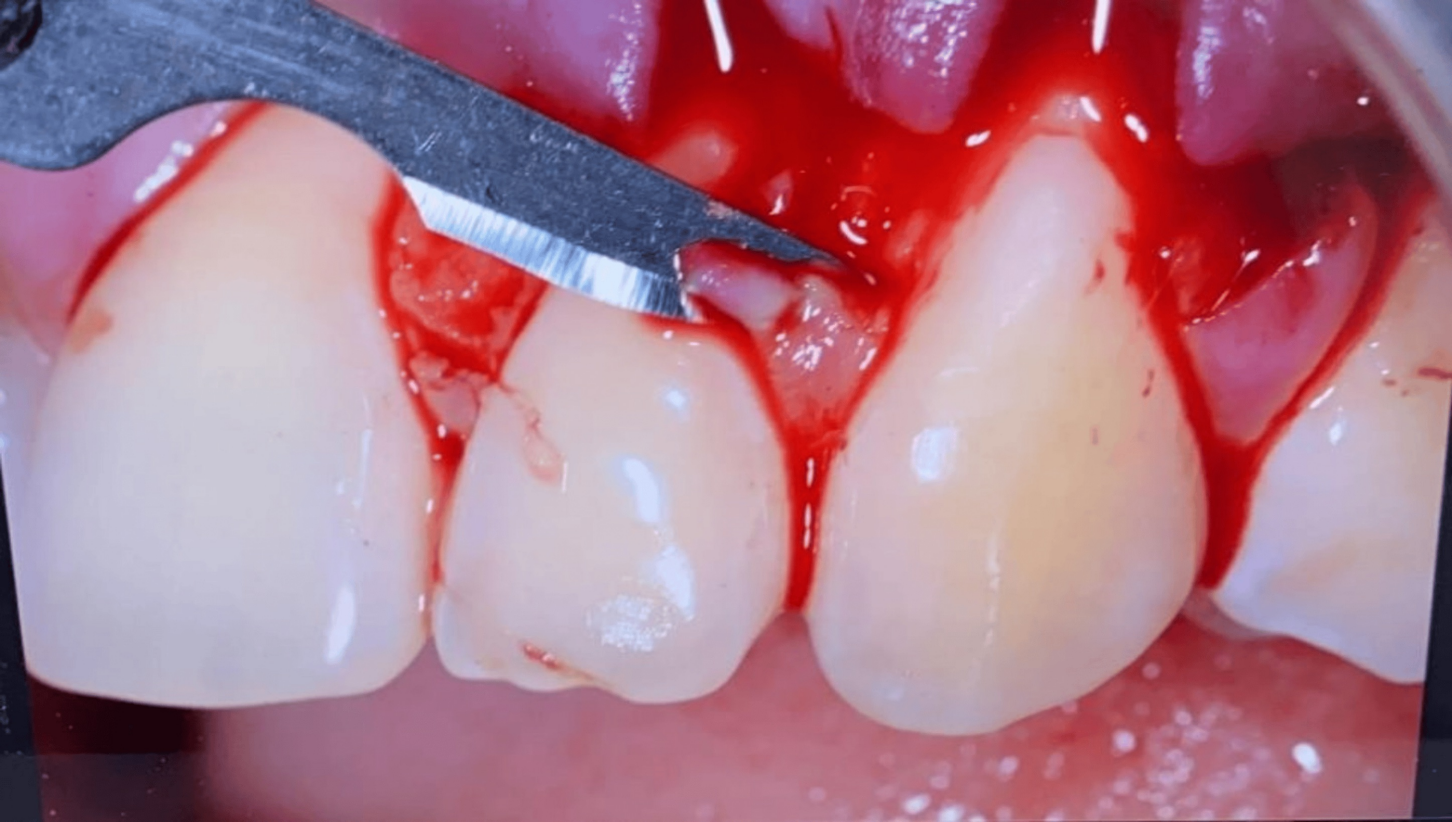
Anatomic papilla de-epithelialization and connective tissue bed preparation.

The anatomic papilla was de-epithelialized to expose connective tissue, creating a bed for flap stabilization, as evidenced in Figure 4. During coronal advancement, each surgical papilla was rotated towards the flap periphery and finally resided at the center of the interproximal area (anatomic papilla) that was previously de-epithelialized. Flap mobilization was adequate when the flap marginal portion passively reached a level coronal to CEJ at every tooth in the surgical area and when the surgical papillae covered the corresponding anatomic papillae.

Root Surface Preparation and Connective Tissue Graft Placement 

**Figure 5:**
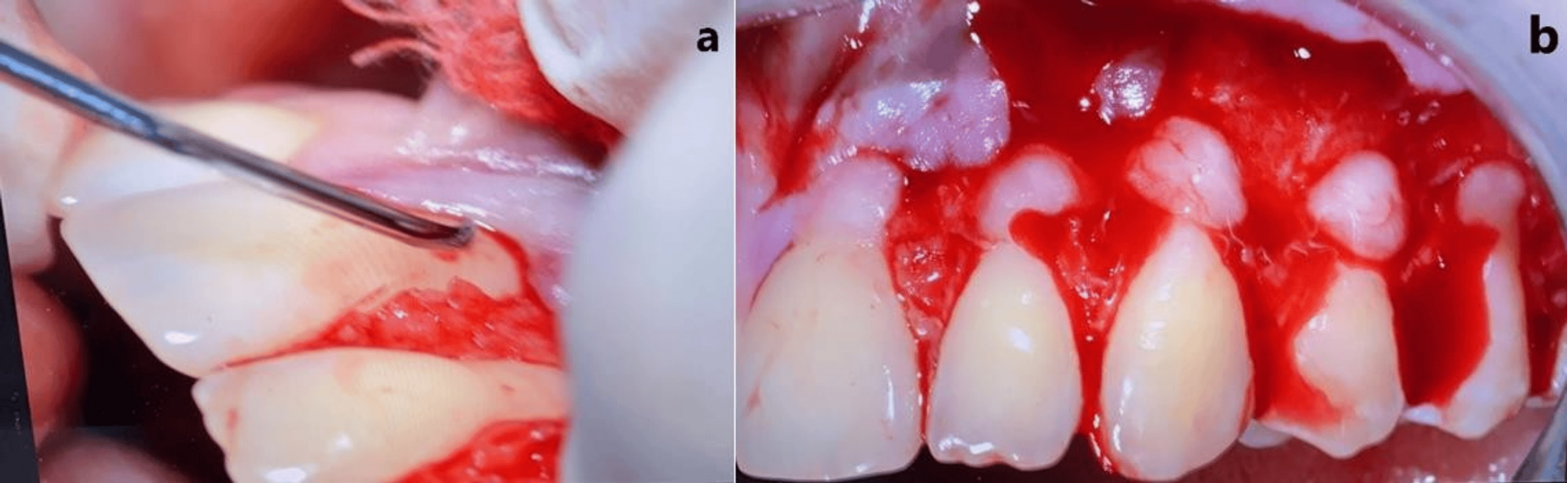
Intraoperative view.

**Figure 6:**
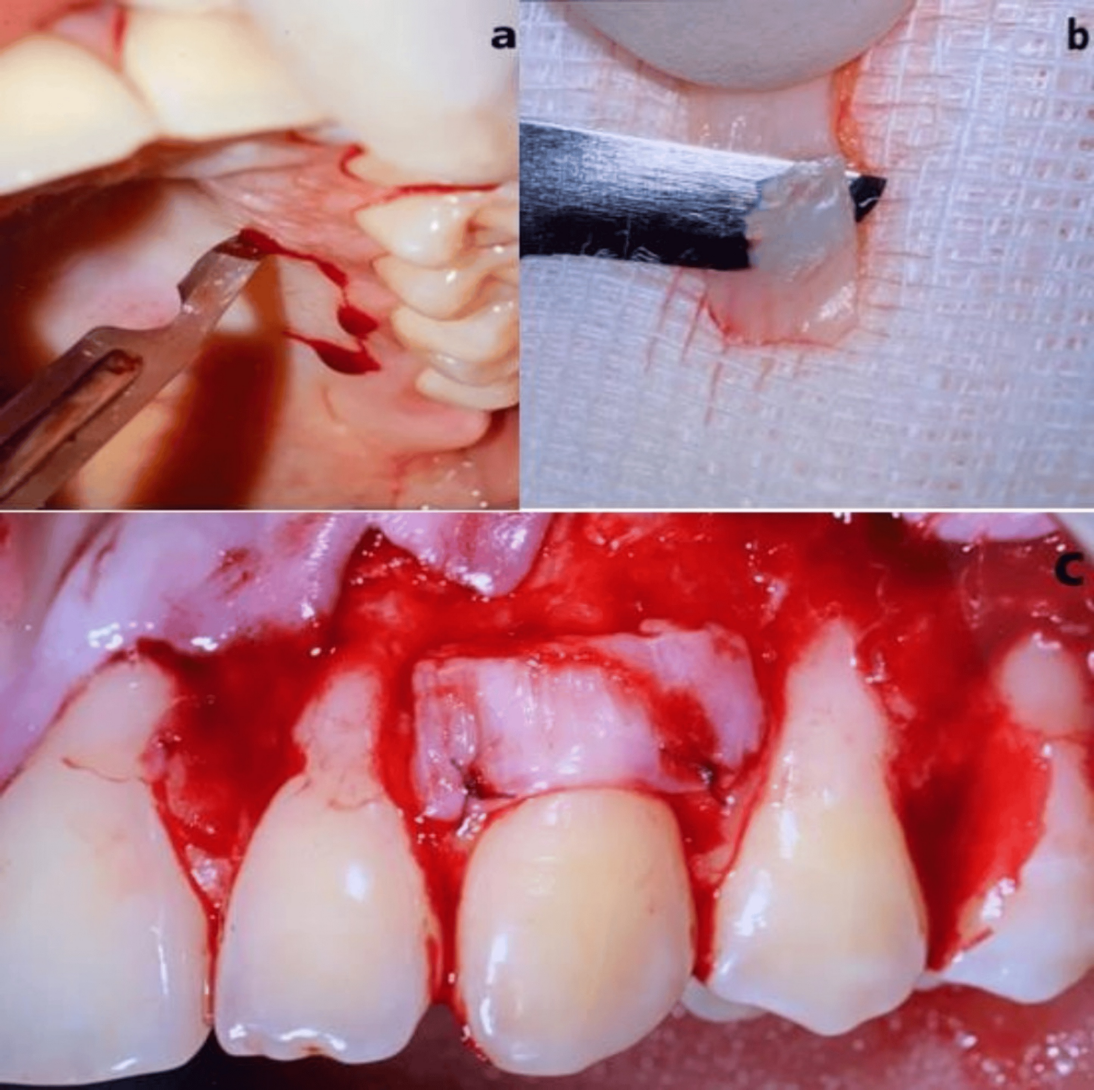
Connective tissue graft placement.

**Figure 7:**
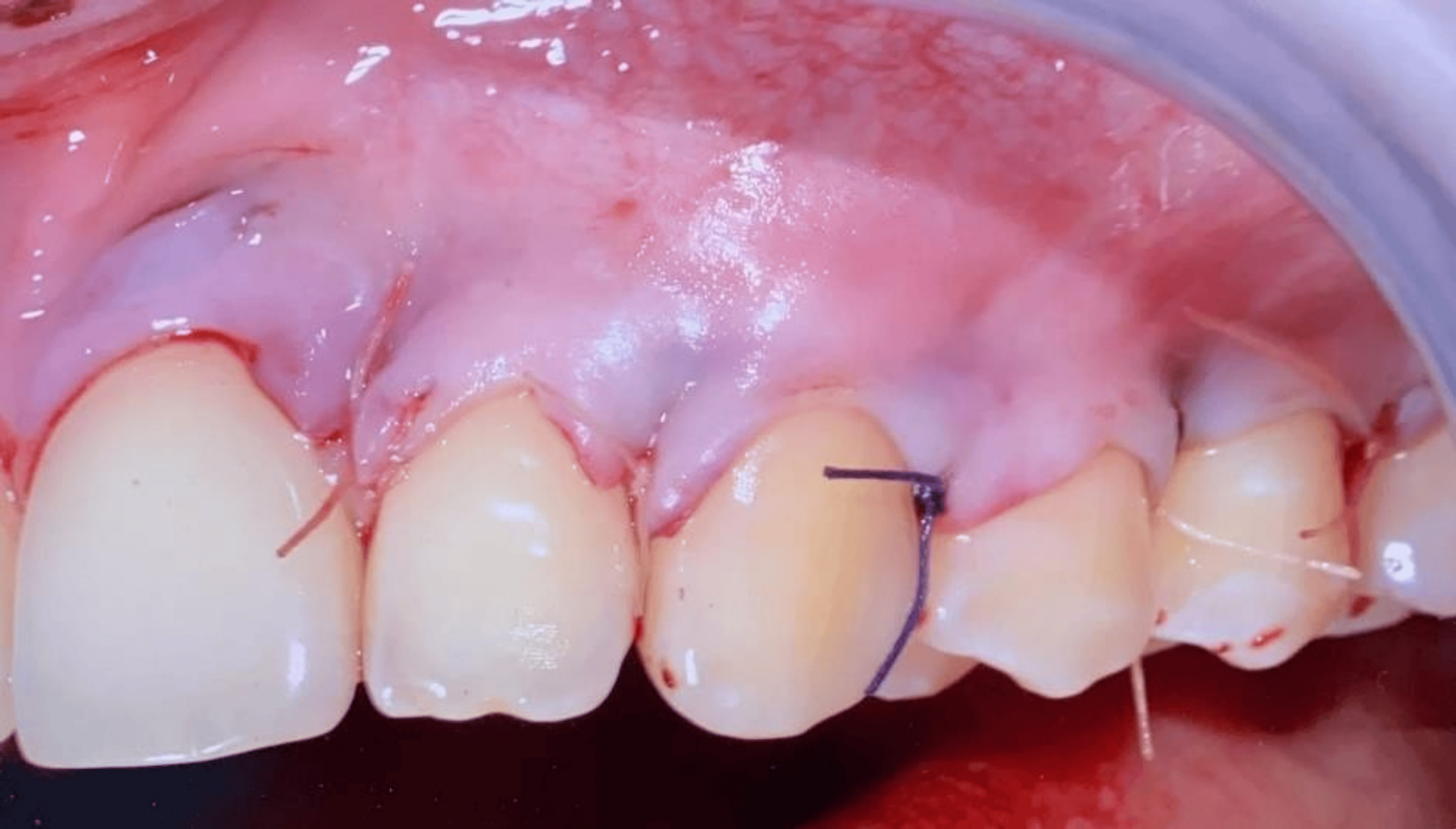
Flap suturing and advancement.

Exposed root surfaces were treated with Gracey curettes, followed by 24% EDTA (Straumann® PrefGel®) for smear layer removal, and rinsed with saline before applying Straumann® Emdogain® (Figure 5). A CTG, harvested using a trap-door technique, was trimmed and secured over the root surfaces with resorbable sutures (Figure 6). Coronal advancement of the flap was achieved, followed by stabilization with 6-0 sling sutures and interrupted sutures [21,24] (Figure 7).

Postoperative Care and Healing 

Postoperative care following the root coverage procedure was prescribed, and the sutures were removed after 14 days. The healing process was uneventful, evidencing recession reduction. KTW and GT increased, and patient-centered outcomes improved, in terms of lower DH and esthetic conditions.

Clinical and patient-assessed outcomes 

**Figure 8:**
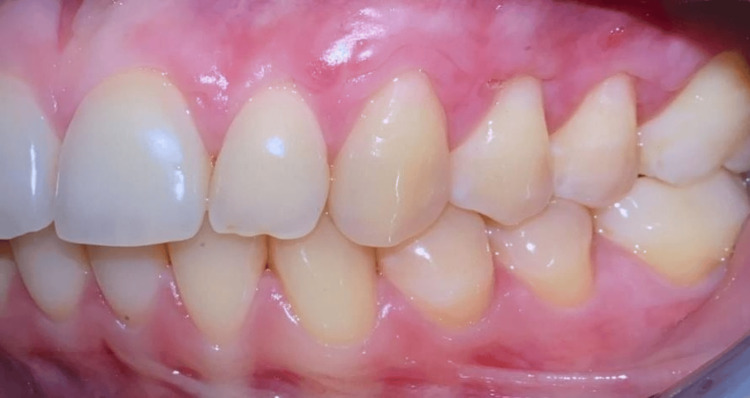
Lateral view of the maxillary left quadrant one month after surgical procedure showing the correction of the mucogingival defects.

**Figure 9:**
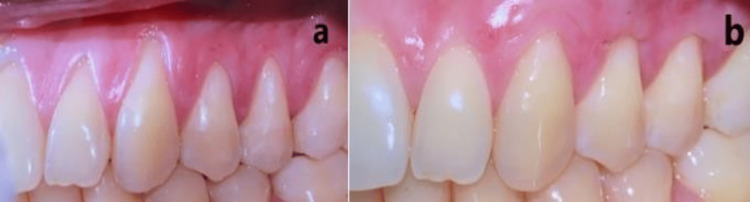
Baseline and postoperative buccal views. (a) Baseline buccal view showing multiple recession defects from 22 to 25. (b) Buccal view one-month post-surgical procedure.

At one-month follow-up, the patient expressed satisfaction with the treatment outcome, as evidenced by improved aesthetics and reduced dentinal sensitivity. A substantial decrease in the depth of gingival recession was noted. Improvement in CAL was noted, indicating reduced tissue loss, as evidenced in Figures 8-9. Since maintenance of good OH was crucial for long-term success, regular OH instructions were provided to the patient. Percentage change was calculated by dividing the difference between the baseline value and the two-month-old value by the baseline value.

Follow-up

The patient was scheduled for regular follow-up appointments to monitor healing and address any concerns. Periodic clinical examinations were conducted to assess the stability of the treatment outcome and identify any potential issues. Table 1 shows clinical measurements at baseline and two-month follow-up.

**Table 1:** Clinical measurements at baseline and two months. PD: probing depth; GR: gingival recession; CAL: clinical attachment level; PI: plaque index; BOP: bleeding on probing; GT: gingival thickness; KTW: keratinized tissue width; DH: dentin hypersensitivity; T0: baseline; T1: 2-months

Clinical Measurements	# 22 (T0)	#22 (T1)	Change (T0-T1)	#23 (T0)	# 23 (T1)	Change (T0-T1)	# 24 (T0)	# 24 (T1)	Change (T0-T1)	# 25 (T0)	# 25 (T1)	Change (T0-T1)
PD (mm)	1	1	0	1	1	0	1	1	0	1	1	0
GR (mm)	2	0.5	-75%	3.5	1	-71.43%	1.5	1	-33.33%	2	1	-50%
CAL (mm)	3	1.5	-50%	4.5	2	-55.56%	2.5	2	-20%	3	2	-33.33%
PI (%)	0	0	-	0	0	-	0	0	-	0	0	-
BoP (%)	0	0	-	0	0	-	0	0	-	0	0	-
GT (mm)	1	1.8	80%	0.8	2	150%	1.3	1.9	46.15%	1.4	1.9	35.71%
KTW (mm)	2.5	2.9	16%	1.5	2.5	66.67%	2	2.5	25%	2	2.5	25%
DH (VAS)	4	0	-100%	4	0	-100%	4	0	-100%	4	0	-100%
Esthetics (VAS)	2	8	300%	2	8	300%	2	8	300%	2	8	300%

Intervention adherence and tolerability

The patient adhered to the prescribed OH regimen and postoperative care instructions. The patient tolerated the surgical procedure and postoperative healing well, with minimal discomfort. No significant adverse events or complications were reported during or after the treatment.

## Discussion

This case report demonstrated the successful management of multiple sites of gingival recession defects using a modified envelope flap technique combined with CTG and EMD. Overall, the clinical case presented here confirmed that more predictable outcomes resulted from the association between CTG and CAF. This could be attributed to the dual blood supply from both the flap and periosteum from the surgical site around the recession, and complete tissue color integration was achieved and treated area texture with the adjacent soft tissues [13,25]. Relevant data showed that the two combinations (CAF + CTG and CAF + EMD) provide better results than CAF alone, and no other therapy provides better results than CAF + CTG in terms of complete root coverage and recession reduction [26]. Moreover, CTG applied at the level of the CEJ prevents shrinkage and improves CAF stability at the time of surgery [20].

Specifically, the surgical approach described here was the modified design of the envelope flap proposed by Zucchelli et al. [23] for the treatment of multiple recession defects to treat continuous recessions simultaneously. The envelope flap was associated with an increased probability of obtaining complete root coverage and with a better postoperative result such as a greater increase in buccal KTW, a more harmonious scalloped, knife-edged outline of all teeth belonging to the quadrant jaw, and the contiguity (the general inability to distinguish the treated area from the adjacent soft tissues), as demonstrated by Zucchelli et al. [21].

The main factors influencing the decision to add a CTG include the lack of keratinized tissue (KT) apical to the root exposure, the need for increased GT, and the presence of a deep abrasion defect [27]. In this present case, the decision taken to use a graft under a CAF was to obtain the best mean root coverage percentage, complete root coverage, and KT gain from short- and long-term outcomes, as evidenced in the literature [24,28-30]. Since graft healing is affected by CTG size, which is determined by the avascular surface area dimensions, large grafts can impair the vascular exchange between the covering flap and the underlying receiving bed, thereby increasing the potential for flap complications, such as dehiscence and unesthetic graft exposure [27,31]. Zucchelli et al. [32] suggested that the reduced apicocoronal CTG dimension and thickness could facilitate graft coverage by the flap, improving esthetic outcomes and reducing patient morbidity with no change in root coverage predictability. The use of CTG modifies the gingival tissue biotype, making it more resistant to traumas, thus reducing the risk of new recessions and eventually providing a more stable long-term outcome [30].

The combination of EMD and CAF + CTG, especially indicated with large root exposure and buccally dislocated root, was adopted to improve attachment quality between the soft tissue and the root, to increase soft tissue thickness (critical factors for the long-term stability of root coverage outcome) and to improve wound healing and the patient’s post-operative comfort [33]. In this respect, Cairo et al. [34] performed a systematic review and found that CAFs with CTGs with and without EMD provided the best esthetic outcomes for root coverage procedures in agreement with Sculean et al. [35].

Several procedures are used for the management of gingival recession defects, representing a significant therapeutic and aesthetic concern in current periodontal practice. Even if the procedure that was adopted in this present case is the gold standard for multiple recession areas [23], each clinical situation should be evaluated to determine the most appropriate surgical approach to achieve the patient’s esthetics expectations. Thus, a meticulous assessment of key anatomical parameters, such as papilla height, keratinized tissue width, periodontal biotype, and vestibule depth, is essential for the surgical decision-making process [24].

## Conclusions

The increasing demand for root coverage emphasizes esthetic outcomes over sensitivity reduction, making gingival recession management a significant focus in modern periodontal practice. Achieving predictable root coverage requires careful consideration of esthetic concerns and patient-centered outcomes, including complete root coverage and soft-tissue variables. This case report highlights the effectiveness of a modified envelope flap technique along with CTG and EMD for treating multiple gingival recessions. While the approach demonstrates promising esthetic and therapeutic outcomes, further studies with larger sample sizes and long-term follow-up are necessary for validation. Furthermore, individual patient factors and defect severity can influence results. Future research is essential to evaluate long-term stability and refine evidence-based surgical techniques for optimal patient care.
